# Assessing the effect of powder characteristics of infant milk on the compressibility of milk powder compression molding

**DOI:** 10.1002/fsn3.3425

**Published:** 2023-05-15

**Authors:** Shaoli Liu, Xuefeng Jiang, Feifan Fu, Tariq Aziz, Guipu Li, Juanjuan Zhao, Sana Shah, Gongnian Xiao, Jinyan Gong, Guanghua He

**Affiliations:** ^1^ School of Biological and Chemical Engineering Zhejiang University of Science and Technology Hangzhou China; ^2^ School of Food and Biological Engineering Jiangsu University Zhenjiang China; ^3^ Institute of Basic Medical Sciences Khyber Medical University Peshawar Pakistan; ^4^ Beingmate (Hangzhou) Food Research Institute Co., Ltd. Hangzhou China; ^5^ Xi'an Yiqian Dairy (Group) Co., Ltd. Xi'an China

**Keywords:** infant milk, particle size, solubility, strength, water content

## Abstract

Infant formula is an important food for those infants who are deprived of breast milk. However, infant formula powder is prone to fly apart, moisture absorption, sticky spoon, and inaccurate measurement. Block infant formula can solve these problems well. In this study, the characteristics (including particle structure morphology, moisture content, particle size, etc.) of infant formula powder were investigated on the compressive strength and solubility of block infant formula after compression molding with infant formula powder as the object. The results showed that the compressive strength and solubility of the block infant formula made from milk powder with a moisture content of 4.75%, particle size larger than 80 mesh, and morphology of compact grape structure appendages were the best. Therefore, milk powder with this characteristic is the most suitable for the preparation of block infant formula. This study provides referenceable experimental data and theoretical basis for the preparation and application of block infant formula.

## Graphical abstract

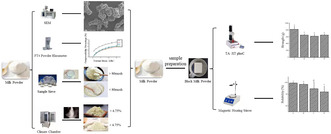

The aim of this research work was to study the effects of powder characteristics, including particle structure, moisture content, and particle size, on the compressive strength and solubility of block milk powder after compression molding.

## 1 INTRODUCTION

Milk is an important food for children and the best source of nutrition for babies is breast milk. World Health Organization (WHO) recommends that infants be solely breastfed for the first 6 months of life. Even though it is not possible to generate the same product as breast milk, several attempts have been made to replicate the nutritional characteristics of breast milk for the normal growth of infants. Infant formulas (IF) are deemed as an effective substitute for breast milk and have been prepared to imitate the nutritional composition of breast milk and should meet the normal physical growth quality and adequate biological quality (Martin et al., 2016). IF powders are dehydrated emulsions consisting of proteins, fat, carbohydrate, vitamins, and minerals which are vital for the infant's nourishment in the absence of breast milk (Murphy et al., 2020). Complimentary follow‐on (FO) formulas can be used as supplementary food when the infant grows, and solid foods are introduced. Several IF and FO powders are made with intact bovine proteins; however, specialized products also exist in it such as IF made with hydrolyzed caseins and whey proteins for infants showing adverse reactions to standard formulations (Maldonado et al., 1998).

At present, infant formula basically exists in the form of powder, and the powder has been improved to a large extent in terms of quick solubility. But the following problems still exist in powder morphology: (1) powder may be flying and scattered during brewing (Shibata & Toyoda, 2010), (2) it is easy to absorb moisture and to agglomerate, sticky spoon in use, which will affect the flushing and bring inconvenience to use (Bhandari & Ho, 2020; Fitzpatrick et al., 2010; Listiohadi et al., 2008), (3) inaccurate measurement, resulting in nutritional deficiencies or excesses that affect the health status of the infant (Jeffs, 1989; Socha et al., 2011; Ziegler, 2011). If milk powder is compressed and molded into block form, it can not only avoid these drawbacks but also improve portability and reduce the difficulty of parenting. However, the production of block infant formula. However, not only must no additives be added to produce lumpy milk powder, but it also requires a certain strength and good solubility. Studies have shown that the powder moisture content affects the powder forming strength by influencing the hardness of the particles and the interparticle forces (Wang et al., 2021).

Powder particle size mainly affects powder molding strength by influencing the contact area between particles, the number of contact points, and the bonding strength (Herting & Kleinebudde, 2008; Sun, 2011). Powder structure morphology affects the strength of powder molding by influencing the manner of interparticle bonding mechanisms and the probability of forming certain bonding mechanisms (Lamešić et al., 2018). Nevertheless, these reports are mainly from the pharmaceutical field, where the pharmaceutical process only needs to consider the effect of powder moisture content, particle size, and structural morphology on molding without focusing on its solubility in water. There are fewer studies in the field of infant formula milk powder. In this study, according to the characteristics and requirements of block infant formula, the effects of moisture content, particle size, and structural morphology of powder on the compressive strength and solubility of block infant formula after compression molding were carried out. The results of the study provide theoretical support for the future processing of block infant formula.

## 2 MATERIALS AND METHODS

### 2.1 Samples and equipment

Four different commercially available brands of infant formula labeled as milk powder A, B, C, and D. Milk Powder A, B, and D come with cow's milk as the main ingredient and C with goat's milk as the main ingredient. Magnetic heating stirrer (79‐1, Jintan Huafeng Instrument Co., Ltd.), electric tablet press (YPD‐20S, Tianjin Hengchuangda Technology Development Co., Ltd., China), climate chamber (BIC‐250, Shanghai Boxun Industrial Co., Ltd.), electric blast dryer (DHG‐9070A, Shanghai Yiheng Scientific Instrument Co., Ltd.), texture analyzer (TA‐XT plusC, Stable Micro Systems), electronic balance (JA1003, Shanghai Liangdan Instrument Co., Ltd.), scanning electron microscope (SEM, SU1510, HITACHI), and powder rheometer (FT4, Freeman Technology) were used.

### 2.2 Milk block production process

4.5 g of milk powder was weighed and put into the mold, the pressure of the tablet press was set at 0.3t, and the pressure holding time was set at 2 s, and then it was compressed to form semifinished in blocks. The semifinished products were put into the artificial climate chamber, where the temperature was 40°C and the relative humidity was 80% for 8 min, and then it was put into the drying oven at 40°C for 30 min (Mansui, 2009).

### 2.3 Observation of the structural morphology of powder particles

Scanning electron microscope analysis was employed to characterize the surface morphologies of the infant formula. The samples were placed on conductive carbon tape and sputter‐coated with a layer of gold prior to taking images. Samples were observed at 5 and 15 kV accelerating voltages.

### 2.4 Air permeability testing

The air permeability of milk powder particles was examined by FT4, keeping the base air flow rate at 2 mm/s and applying normal positive pressure changes eight times, in the order of 1, 2, 4, 6, 8, 10, 12, and 15 Kpa. The pressure drop was measured only once after each positive pressure change for a total of eight measurements (Freeman, 2007; Gnagne et al., 2017; Lefebvre et al., 2020). The permeability was characterized by the size of the pressure drop, and the larger the pressure drop, the weaker the permeability (Dipika et al., 2018).

### 2.5 Compressibility testing

The compressibility of the milk powder particles was examined by FT4, and the applied normal stress was increased continuously in the order of 0.5, 1, 2, 4, 6, 8, 10, 12, and 15 Kpa. The compressibility ratio was measured only once after each pressure increase for a total of nine measurements (Freeman, 2007; Gnagne et al., 2017; Lefebvre et al., 2020). The compressibility was characterized by the size of the compression ratio, and the larger the compression ratio, the better the compressibility.

### 2.6 Preparation of samples with different moisture contents

4.5 g of milk powder A was laid flat in a weighing dish and placed in a climatic chamber humidified at a temperature of 20°C and humidity control of 30%rh, 40%rh, 50%rh, and 60%rh, respectively. The milk powder samples A_0_–A_4_ with different moisture contents were weighed every 1 h until a constant weight was obtained (Koç et al., 2010). The moisture content of the milk powder samples was also tested according to GB 5009.3, after which the block infant formula was prepared according to Section 2.2.

### 2.7 Preparation of samples with different particle sizes

Milk powder A was sieved at 120–140 mesh, 100–120 mesh, 80–100 mesh, 60–80 mesh, and >60 mesh to obtain five milk powder samples with different particle size intervals, after which block infant formula was prepared according to Section 2.2 (Jiang et al., 2011; Koç et al., 2010; Schuck et al., 2008).

### 2.8 Block infant formula compressive strength testing

The compressive strength of block infant formula was tested using a texture analyzer as follows: Set TPA test conditions, using A/MORS blade probe, probe speed 2 mm/s before test, 0.1 mm/s during test, 2 mm/s after test, loading distance 3 mm, dwell time 5 s, test pressure 5 g (Jiang et al., 2011; Sun et al., 2007). In the time–pressure curve, the peak value is used as the compressive strength of the block infant formula (Agrahar‐Murugkar et al., 2015). Two pieces of finished products were selected, each piece of three different parts of the compressive strength was tested and the average value was taken.

### 2.9 Solubility determination

Place the block of milk powder at 50°C, 150 mL of warm water, 1,957× *g* for stirring. After stirring for 180 s, filter through a 100‐mesh filter. The residue was dried in the oven until constant weight was obtained. Repeat three times and take the average value to calculate the dissolution rate (Fyfe et al., 2011; Jiang et al., 2021).

### 2.10 Statistical analysis

Statistical analyses and data analysis of single factor variance (ANOVA) were conducted using IBM SPSS Statistics 22 software.

## 3 RESULTS AND DISCUSSION

### 3.1 Morphology of different milk powder samples

**FIGURE 1:**
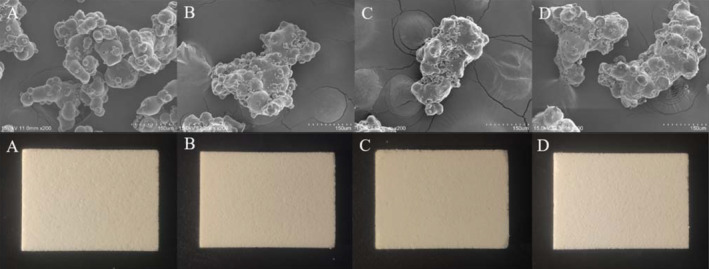
Observation of milk powder samples (A–D) by SEM and block milk made from different milk powders.

It can be seen from Figure 1, milk powder A particle morphology structure is in the form of tight grape‐like structure attachment, single particle size is relatively uniform, and the surface is smooth. There are certain angles between the particles, which is easy to form mechanical interaction between the particles during compression molding. At the same time, the force between the particles is conducive to compression molding. The shape structure of milk powder B–D particles is an onion structure polymer, and the size of individual particles is not uniform. Many fine particles adhere to the surface of the polymer. Compared with milk powder A, the possibility of mechanical interaction between particles during compression molding is lower, and only interparticle force can bind the particles to form. Visually, there is no major difference among the pressed blocks of milk powder.

### 3.2 Air permeability and compressibility of different milk powder samples

**FIGURE 2:**
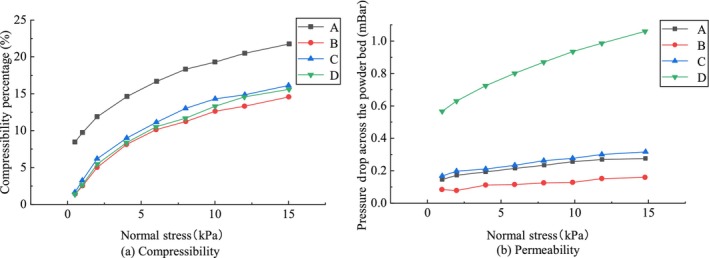
Comparison of (a) compressibility and (b) air permeability among samples (A–D).

Among the four milk powder samples, the compression ratio of milk powder A was higher than that of milk powder B–D (Figure 2a), while the compression ratio curves between milk powder B–D were similar, indicating that the deformation of milk powder A particles was larger than that of milk powder B–D under the action of the same external force. As can be seen from Figure 2b, the air permeability strength is D < C < A < B. Air permeability reflects the degree of interparticle bonding. The poorer the permeability, the higher the degree of interparticle bonding and the greater the interparticle forces, which means that the solubility of the milk powder after being made into blocks is also poor. Because in the compression process, poorly permeability samples are also less capable of exhausting air and tend to leave air bubbles in the molding body. This result suggests that sample D is more suitable to be made into block infant formula.

### 3.3 Effect of particle structure morphology on the solubility and compressive strength of block infant formula

**FIGURE 3:**
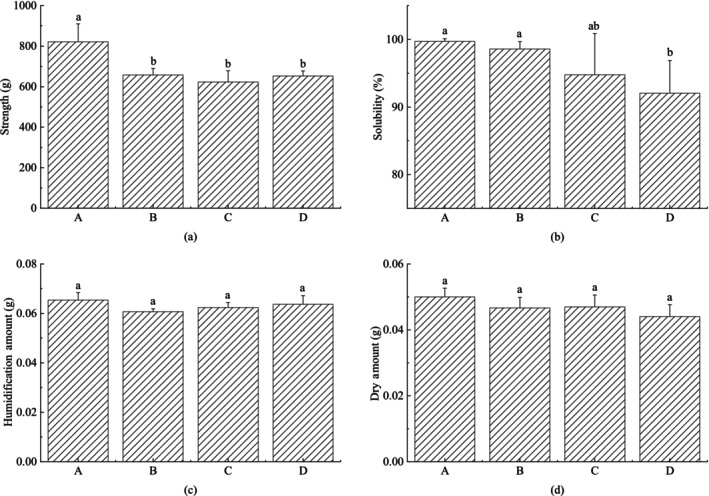
Effect of different milk powder particle structure morphology on the characteristics of block milk powder. (a) Compressive strength of the samples. (b) Solubility of the samples. (c) Humidification amount: the amount of water absorbed by the block of milk powder semi‐finished products in the humidification process. (d) Drying amount: the amount of water evaporated during the drying process of block milk powder. Different letters above the column indicate significant differences in the different samples (*p* < .05). Error bars indicate standard error of means (*n* = 3).

The milk powder particle morphology affects the solubility and compressive strength of the block infant formula. The results showed that the compressive strength of block infant formula A was higher than the rest of the block infant formula, and the strength of the rest of the block infant formula was similar (Figure 3a). It is presumed that the compression ratio of milk powder A is higher than the remaining three milk powders. Under the same pressure, milk powder A particle deformation, the contact area between the particles, and the force between the particles are greater. In the meantime, milk powder A particles are easy to mechanically lock together, so the strength of block infant formula A is higher. Milk powder B–D permeability decreases in turn, the interparticle viscosity increases which will increase the compressive strength. Meanwhile, the formation of internal bubbles also increases during compression, which will reduce the compressive strength. The two factors make the compressive strength similar.

From sample A to sample D, the solubility decreases successively (Figure 3b). Compared with the other three kinds of milk powder with onion structure, milk powder A with tight grape structure has better particle dispersion and uniform size. During compression, large pores can be formed between the particles, which is convenient for liquid penetration and dissolution. Therefore, it has good solubility. Because the air permeability of B, C, and D milk powder decreases, the stickiness between particles and the binding force increase, resulting in a decrease of solubility.

The moisture content absorbed during the humidification step (humidification amount) and the moisture content evaporated during the drying step (drying amount) can have an effect on the surface layer strength of the block infant formula. As can be seen from Figure 3c,d, the humidification and drying amounts of block infant formula made from different milk powders are basically the same. It shows that the particle morphology does not affect the humidification and drying amounts, so the block infant formula surface strength is approximately equal.

In conclusion, milk powder with compact grape structure polymer is more suitable for the preparation of block milk powder, compression ratio, and air permeability can assist in the evaluation of powder structure.

### 3.4 Effect of different moisture contents of milk powder on the solubility and compressive strength of block infant formula

**FIGURE 4:**
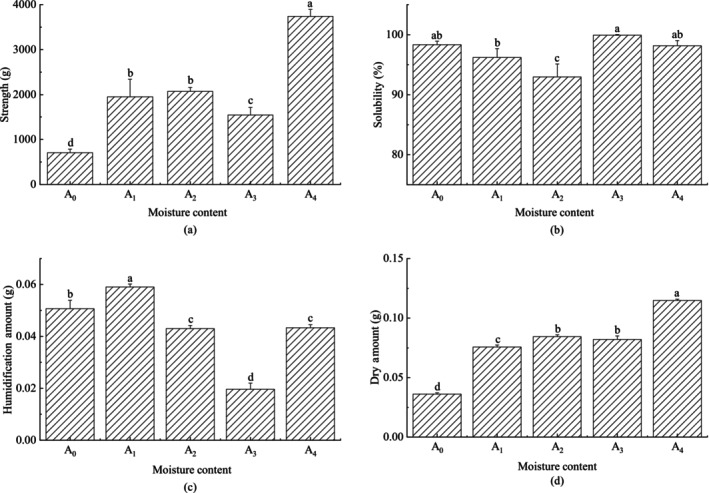
Effect of different milk powder particle structure morphology on the characteristics of block milk. A_0_‐A_4_ represents the moisture content of milk (A_0_: 2.90%; A_1_: 3.10%; A_2_: 4.37%; A_3_: 4.75%; A_4_: 6.00%). (a) Compressive strength of the samples. (b) Solubility of the samples. (c) Humidification amount: the amount of water absorbed by the block of milk powder semi‐finished products in the humidification process. (d) Drying amount: the amount of water evaporated during the drying process of block milk powder. Different letters above the column indicate significant differences in the different samples (*p* < .05). Error bars indicate standard error of means (*n = 3*).

The effect of different milk powder moisture contents on the characteristics of block infant formula has been shown in Figure 4. The moisture content of raw milk powder is closely related to the compressive strength and solubility of block infant formula after compressed type of milk powder. The results showed that the drying amount of block infant formula made of raw material A_0_ is lower than the humidifying amount, while the drying amount of block infant formula made of sample A_1_–A_4_ is greater than the humidifying amount. As the moisture content of milk powder increased, the compressive strength of the compressed molded block increased simultaneously, but the solubility gradually decreased. When the moisture content continued to increase to 4.75% (A_3_), the compressive strength decreased slightly, but the solubility increased rapidly. When the moisture content further increased to 6.00% (A_4_), the compressive strength increased rapidly, and the solubility decreased slightly.

In the preparation of block infant formula, the purpose of humidification is to dissolve the milk powder particles in the adjacent parts of the surface to form a joint, and the purpose of drying is to solidify the milk powder with adhesion on the surface to form a solid bridge. Reasonable humidification and drying processes can obtain better compressive strength and solubility of block infant formula. There was a study (Mansui, 2009) that believed that the surface compressive strength of block infant formula was affected by the amount of humidification and drying. In general, the amount of humidification and drying were equal, and the thickness range of the surface forming a solid bridge can be enlarged by increasing the same amplitude of these two factors, thus increasing the compressive strength of the surface layer of lumpy milk powder. The moisture content of the raw material will affect the humidification and drying amount and will affect the thickness of solid bridge formed on the surface. Appropriate increase in the moisture content of raw materials is conducive to promoting the thickness of the surface layer to form a solid bridge and improve the compressive strength, in addition to reducing the force between the particles and improving solubility. However, it is also believed that with the increase of moisture content of particles, the initial stage is easy to form and the compressive strength increases, and then the water film formed on the surface of particles will hinder the interparticle force and reduce the compressive strength (Ali et al., 1996; Parkash et al., 2017; Wang et al., 2021).

Therefore, it is crucial to control the moisture content of raw milk powder. According to the results of this experiment, the amount of water content of raw powder milk is controlled at 4.75%, which is good for the preparation of block infant formula.

### 3.5 Effect of particle size on compressive strength and solubility of block infant formula

**FIGURE 5:**
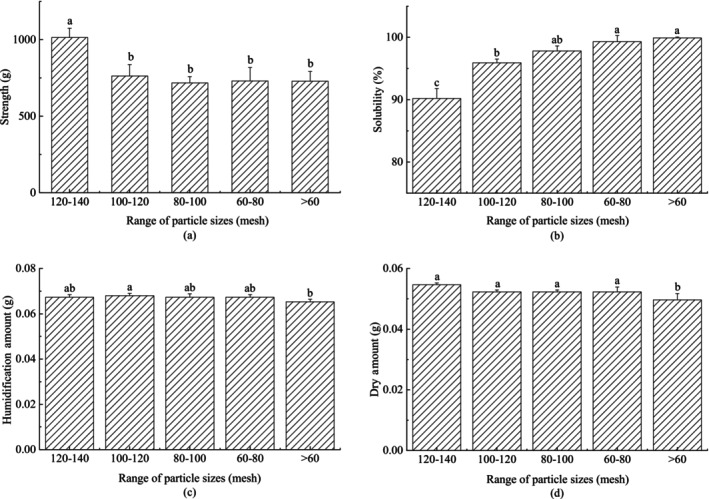
Effect of different milk powder particle size on the characteristics of block milk. (a) Compressive strength of the samples. (b) Solubility of the samples. (c) Humidification amount: the amount of water absorbed by the block of milk powder semi‐finished products in the humidification process. (d) Drying amount: the amount of water evaporated during the drying process of block milk powder. Different letters above the column indicate significant differences in the different samples (*p* < .05). Error bars indicate standard error of means (*n = 3*).

The effect of different milk powder particle sizes on the characteristics of block infant formula has been shown in Figure 5. It can be seen from Figure 5a, the compressive strength of block infant formula has a downward trend with the increase of milk powder particles, and then tends to be gentle. The solubility of block infant formula improved with the increase of milk powder particles (Figure 5b). The compressive strength of block infant formula gradually decreases with the increase of particles, and the decreasing trend is gradually slowing down. The changes in the humidification and drying amounts were not significantly related to the particle size of milk powder (Figure 5c,d), indicating that the particle size of milk powder did not affect the thickness of the solid bridge formed on the surface of block infant formula, that is, the particle size did not affect the strength of the surface layer of block infant formula, but would have an effect on the interparticle forces within the block infant formula.

**FIGURE 6:**
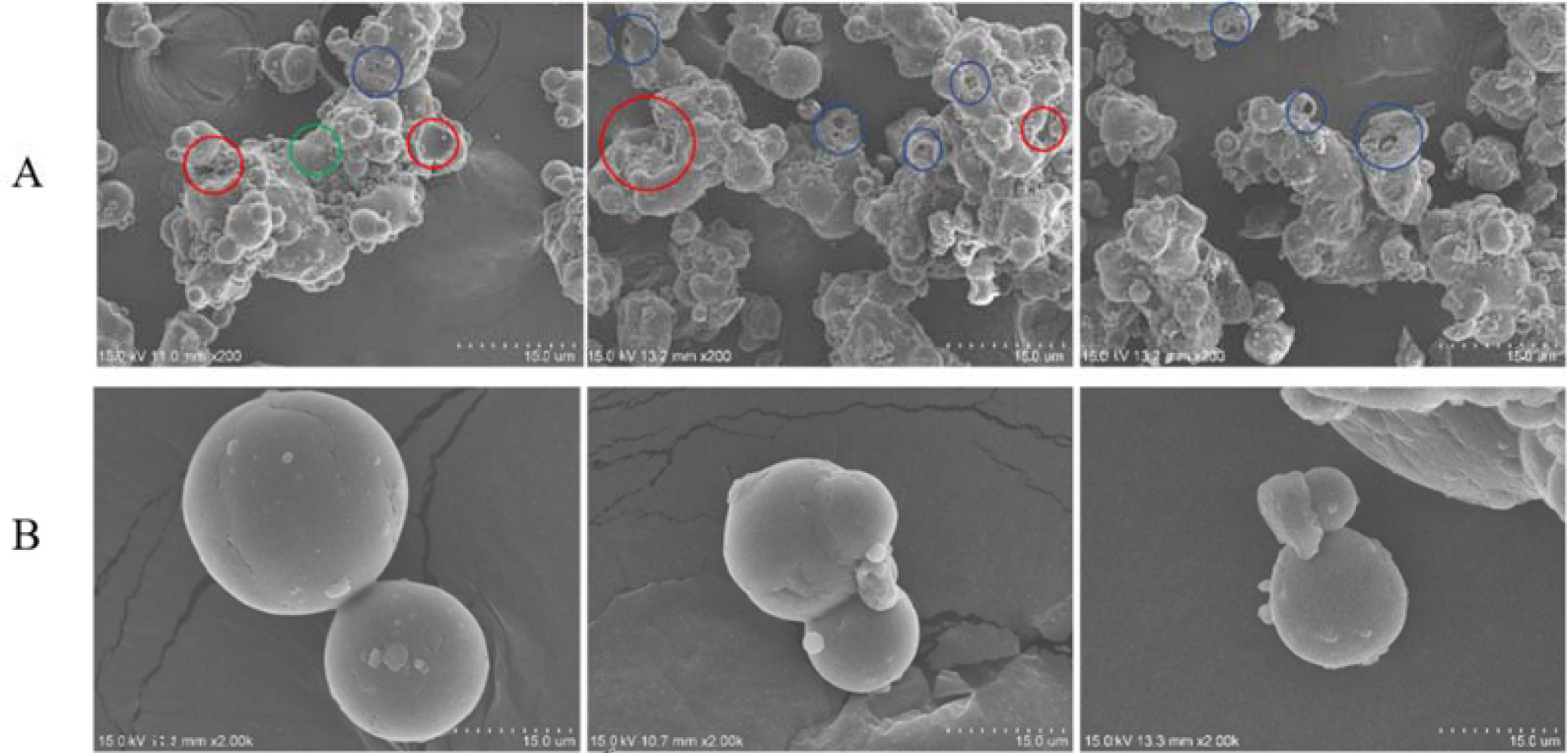
Comparison of milk powder particle size and defects. (A) Large particle, (B) small particle, red circle: dent, green circle: crack, blue circle: hole.

By comparing the top and bottom of Figure 6, it can be seen that the surface of large particles has multiple defects, such as holes, dents, cracks, etc. While the surface of small particles is smooth, basically showing round particles without obvious defects. Therefore, the larger the particle, the more defect points on the particle. The compressive strength of block infant formula gradually decreases with the increase of particles. It is analyzed that the contact points and binding area between particles decrease due to the increase of particles, resulting in the decline of the compressive strength of block milk powder, which is consistent with the reports of Herting and Kleinebudde (2008), Sun (2011) and Yingmeng et al. (2019). It is also reported that the larger the particles are, the more defect points the particles may have and the smaller the particle strength is. The larger the particles are, the easier to break under the same pressure conditions, which may also be the reason for the decrease in compressive strength of block infant formula (Herting & Kleinebudde, 2008; Sun, 2011; Yingmeng et al., 2019). Although a small number of particles break under the same pressure, adding the number of contact points between particles, the vast majority of particles are still large and have few contact points, thus the solubility continues to increase. In summary, milk powder with particle size greater than 80 mesh is more suitable for making block infant formula.

## 4 CONCLUSION

The correlation between the compressive strength and solubility of milk powder after compression molding was studied by the structure, compressibility, permeability, moisture content, and particle size of different milk powder brands. Grape‐structured milk powder particles are better dispersed, uniform in size, and have larger pores between the particles when compressed, which facilitates permeation solubility and therefore higher solubility. Therefore, the milk powder with compact grape structure epimer is more suitable for the preparation of block infant formula. The moisture content of raw milk powder controlled at about 4.75% is beneficial to the preparation of block infant formula. The larger the milk powder particles, the smaller the compressive strength and the greater the solubility of the block infant formula after compression molding. This study provides a reference for further optimization to determine the optimal moisture content range.

## AUTHOR CONTRIBUTIONS

**Shaoli Liu:** Conceptualization (equal). **Guipu Li:** Data curation (equal). **Jinyan Gong:** Investigation (equal). **Guanghua He:** Validation (equal). **Xuefeng Jiang:** Conceptualization (equal); methodology (equal); software (equal); validation (equal). **Tariq Aziz:** Investigation (equal); writing – original draft (equal). **Feifan Fu:** Funding acquisition (equal); methodology (equal); resources (equal). **Juanjuan Zhao:** Formal analysis (equal); methodology (equal). **Sana Shah:** Data curation (equal); validation (equal). **Gongnian Xiao:** Investigation (equal); software (equal).

## CONFLICT OF INTEREST STATEMENT

The authors declare that they have no conflict of interest.

## Data Availability

All the data generated in this research work have been included in the manuscript.
